# Collagen-Gold Nanoparticle Conjugates for Versatile Biosensing

**DOI:** 10.3390/s17020378

**Published:** 2017-02-15

**Authors:** Sarah Unser, Samuel Holcomb, ReJeana Cary, Laura Sagle

**Affiliations:** Department of Chemistry, College of Arts and Sciences, University of Cincinnati, 301 West Clifton Court, Cincinnati, OH 45221-0172, USA; unsersa@mail.uc.edu (S.U.); holcomsj@mail.uc.edu (S.H.); rejeana.m.cary@gmail.com (R.C.)

**Keywords:** collagen, gold nanoparticles, optical biosensors, plasmonic biosensing, localized surface plasmon resonance, plasmonic coupling

## Abstract

Integration of noble metal nanoparticles with proteins offers promising potential to create a wide variety of biosensors that possess both improved selectivity and versatility. The multitude of functionalities that proteins offer coupled with the unique optical properties of noble metal nanoparticles can allow for the realization of simple, colorimetric sensors for a significantly larger range of targets. Herein, we integrate the structural protein collagen with 10 nm gold nanoparticles to develop a protein-nanoparticle conjugate which possess the functionality of the protein with the desired colorimetric properties of the nanoparticles. Applying the many interactions that collagen undergoes in the extracellular matrix, we are able to selectively detect both glucose and heparin with the same collagen-nanoparticle conjugate. Glucose is directly detected through the cross-linking of the collagen fibrils, which brings the attached nanoparticles into closer proximity, leading to a red-shift in the LSPR frequency. Conversely, heparin is detected through a competition assay in which heparin-gold nanoparticles are added to solution and compete with heparin in the solution for the binding sites on the collagen fibrils. The collagen-nanoparticle conjugates are shown to detect both glucose and heparin in the physiological range. Lastly, glucose is selectively detected in 50% mouse serum with the collagen-nanoparticle devices possessing a linear range of 3–25 mM, which is also within the physiologically relevant range.

## 1. Introduction

In recent years, the unique optical properties of noble metal nanoparticles—such as gold and silver—have grasped the attention of researchers for many applications including drug therapy [1], imaging [2], catalysis [3], and biosensing [4,5,6]. Rapid detection, low cost, facile functionalization, and the ability to interface with other on-chip devices that require low sample volume have made these metallic nanoparticles popular for clinical and point-of-care use. Biosensing using plasmonic nanoparticles is often achieved through the sensitivity of the localized surface plasmon resonance (LSPR) to changes in the refractive index at the nanoparticle surface where biological molecules bind. In addition, the interaction of the nanoparticles with a biological molecule can affect the spacing between nanoparticles, altering the plasmonic coupling between the two nanoparticles, which often results in large LSPR shifts and increased sensitivity. Although strides have been made in recent years towards the development of commercial devices which can be used with complex clinical samples, biofouling of the nanoparticle surface with unwanted species in complex biological solutions, such as blood and urine, remains a critical problem [4,5]. Biological molecules, such as enzymes, circumvent the problem of biofouling through an active site which possess shape complementarity and specific molecular interactions for a given ligand [7,8]. Thus, incorporating plasmonic nanoparticles—which are sensitive to their environment—into a biological scaffold which is capable of greater selectivity, and at the same time capable of interacting with targets in a biocompatible manner is a promising approach.

Utilizing biocompatible scaffolds at the nanoscale for functional biomaterials is of broad interest to researchers from many areas of science [9]. Extensive work has been done to develop functional DNA nanoarchitectures that have been integrated with metallic particles [10,11]. Specifically, DNA plasmon ruler systems have been well characterized as sensitive and selective nanosensors that tether nanoparticles together using DNA to probe changes in plasmonic coupling to detect DNA hybridization [12,13], DNA binding proteins [14], and DNA cleaving enzymes [15,16] by monitoring changes in plasmonic coupling. While these studies have demonstrated the sensitivity and selectivity of plasmonic DNA based systems, the biomolecular interactions between DNA and its substrates are limited. The specific ligand binding sites and reactive surfaces of proteins offer the possibility to overcome this limitation by providing diverse functionality compared to DNA nanosystems. Recently, the idea of using proteins as biological scaffolds to study the binding of small molecules through protein conformational changes has emerged in the literature. Van Duyne and co-workers demonstrated this by tethering the protein calmodulin onto silver nanotriangles patterned on glass, then monitored LSPR shifts from the large conformational changes of Calmodulin during ligand association and dissociation for label-free, specific, calcium ion sensing [17]. Large conformational changes, up to 156 nm, in the structure of the extracellular matrix protein fibronectin have been demonstrated when adhering to gold nanotriangles in the presence of live MDCKII cells to monitor in vivo protein conformational changes [18]. While these studies have expanded our ability to detect various biological targets that are otherwise problematic, these studies are limited to proteins that show large conformational changes in addition to refractive index changes, which are considerably smaller than changes observed using plasmonic coupling [19]. Thus, a technique which harnesses the specific reactivity of a protein—while also monitoring plasmonic coupling—would be ideal. 

Collagen is the most abundant extracellular matrix (ECM) protein comprising 30% of extracellular matrix proteins, functioning as both a structural component and signaling through biomolecular interactions. The structural nature of collagen makes it both rigid and a naturally existing protein scaffold, therefore, it is an ideal protein target for a protein-nanoparticle scaffold. Collagen’s presence in the ECM results in a protein that interacts with many diverse biomolecules including proteins [20,21], sugars [22], proteoglycans [21], polyphenols [23], and drug molecules [24], making it an ideal model system for selectively probing a number of molecular interactions. By combining a structurally stable protein with plasmonic nanoparticles, minimal loss of structure and function was expected, once again making collagen an excellent model system for probing biomolecular interactions.

**Scheme 1:**
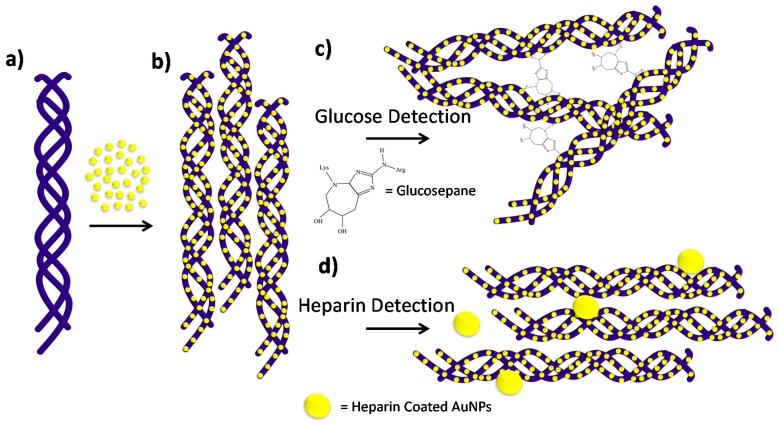
The two sensing schemes addressed in this work using collagen-nanoparticle conjugates. The native collagen before the AuNPs are added (**a**); the collagen after it has been coated in AuNPs forming a collagen-nanoparticle scaffold (**b**); in order to carry out biosensing measurements of glucose, the collagen nanoparticle scaffold is crosslinked by glucose after it has been incubated at 35 °C and the covalent product glucosepane has formed (**c**); lastly, the binding interactions between the collagen-nanoparticle scaffold and the heparin coated 80 nm gold nanoparticles are used to detect heparin (**d**).

In this work, we present a versatile and selective collagen gold nanoparticle construct for biosensing, mediated through plasmonic coupling of gold nanoparticles bound to the collagen fibrils. By integrating collagen fibrils with negatively charged citrate capped gold nanoparticles, a functional plasmonic protein scaffold is presented, see Scheme 1. Harnessing the diverse functions of collagen, the protein-nanoparticle scaffold was used to detect its specific interactions with glucose and heparin through plasmonic coupling. Glucose was directly detected through the formation of non-enzymatic covalent cross-links between neighboring collagen fibrils. As the fibrils are cross-linked together, the nanoparticles on the surface interact through plasmonic coupling, and glucose was detected via the naked eye and a UV-Vis spectrometer, effectively sensing 1–20 mM glucose with a limit of detection of 0.18 mM glucose. A competition assay was then developed for the detection of heparin by first incubating the nanoparticle scaffold with a sample containing heparin, followed by incubation with heparin-coated gold nanoparticles. When the concentration of heparin is in excess, the heparin binding sites on collagen are saturated, which produces a small shift upon addition of heparin-coated nanoparticles. In contrast, at low concentrations of heparin, there are free heparin binding sites, which produce significantly larger shifts with addition of heparin-coated nanoparticles. This competition assay yielded a linear range for heparin detection from 5 to 100 μM, which is within the physiologically relevant range. Lastly, the glucose assay was tested in 50% mouse serum and demonstrates a glucose concentration dependence from 3 to 50 mM glucose in the presence of heat.

## 2. Methods

### 2.1. Materials

Rat tail collagen at a concentration of 2 mg/mL in 0.1% acetic acid was purchased from Serva (Heidelberg, Germany). Monobasic sodium phosphate, dibasic sodium phosphate, sodium chloride, potassium chloride, and d-dextrose were all purchased from Fisher Scientific (Waltham, MA, USA). Amine-PEG-Thiol (MW = 5000) was purchased from Nanocs, Inc. ((New York, NY, USA). Heparin sodium salt from porcine intestinal mucosa (Sigma Aldrich, St. Louis, MO, USA) and phosphotungstic acid (PTA) was purchased from Sigma Aldrich (St. Louis, MO, USA). 80 nm gold colloids were purchased from Ted Pella (Redding, CA, USA) and chloroauric acid, HAuCl_4_, (Spectrum) was purchased from Fisher Scientific.

### 2.2. Gold Nanoparticle Synthesis

Preparation of gold nanoparticles was carried out using the reverse Turkevich method [25]. Briefly, a solution of 25.4 mM of sodium citrate is boiled. Once the solution is brought to a boil, HAuCl_4_ was added to a final concentration of 0.254 mM HAuCl_4_. A color change from gray, to pink, to red was observed, heat was reduced and the solution was mixed for 10 min. Finally, the colloidal suspension was removed from heat and allowed to cool. 

### 2.3. Collagen-Nanoparticle Conjugate Preparation

Dilute and soluble rat tail collagen fibrils were prepared by diluting the acidified concentrated stock solution from 2 mg/mL to 0.004 mg/mL in a low ionic strength PBS stock solution. The low ionic strength of the PBS solution was used in order to prevent nanoparticle aggregation in future dilutions. Low ionic strength PBS was prepared using 51.3 mM sodium chloride, 2.7 mm potassium chloride, 10.1 mM dibasic potassium phosphate, followed by a pH adjustment to 7.2 using 1 M sodium hydroxide, and incubated at 37 °C for 12 h. The diluted collagen solution was then removed from heat and allowed to cool to room temperature. The gold nanoparticles that were synthesized using the reverse Turkevich method were added to the collagen fibril solution in a 3:5 ratio, and incubated at room temperature for 30 min on the benchtop. After 30 min, a UV-Visible spectral measurement was taken. 

### 2.4. Sample Characterization

LSPR measurements were taken in transmission mode using a USB-2000+ UV-Vis spectrometer powered by an HL-2000-HP tungsten halogen lamp and configured with fiber optic cables. Frequency and intensity values for the individual plasmon resonances were obtained by fitting the peaks of interest to a Gaussian function using the Origin 9.0 software (OriginLab, Inc., Northampton, MA, USA). Transmission electron microscopy (TEM) studies were carried out using a JEOL 1230 (JEOL Ltd., Akishima, Tokyo, Japan) at 80 eV, and images were taken using an AMT Advantage Plus 2 × 2 k digital camera. TEM samples were prepared on ultrathin carbon type-A, 400 mesh Cu grids coated with formvar (Ted Pella, Redding, CA, USA) by micropipetting 10 μL of sample onto the grid surface, followed by wicking away the excess solution. Samples were all stained using 1% phosphotungstic acid (PTA) by micropipetting 10 μL of 1% PTA onto the sample on the grid and allowing the stain to sit for 30 s before wicking away the excess solution. All circular dichroism (CD) measurements were taken on a Jasco J-815 CD spectrometer (Jasco Inc., Easton, MD, USA) and a 10 mm path length quartz liquid cell.

### 2.5. Glucose Detection

d-glucose was added in the desired concentration to each individual collagen-nanoparticle conjugate sample, and incubated in a water bath at 35 °C for six hours. Samples were then removed from heat and allowed to cool to room temperature. Once the samples cooled to room temperature final UV-Vis spectral (LSPR) measurements were taken. Kinetic measurements were carried out by incubating a high volume of collagen sample at a given glucose concentration, 0 and 10 mM glucose, in a water bath at 35 °C. Aliquots were then removed at specific time intervals and UV-Visible measurements were taken at these time points.

### 2.6. Mouse Serum Glucose Detection Assay

The collagen-nanoparticle conjugates were tested against 50% mouse serum. The samples were prepared by diluting mouse serum by half in low ionic strength PBS buffer. Collagen-nanoparticle arrays are added to the mouse serum in a 1:1 ratio. An initial LSPR measurement was taken of the mixture of mouse serum and collagen-nanoparticle conjugates. d-Glucose was then added to the solution at a concentration of 50 mM. The solution was then incubated at 70 °C for 30 min, removed from heat, and a final UV-Vis spectral (LSPR) measurement was taken. 

### 2.7. Heparin Detection

Heparin 80 nm gold nanoparticles are formed by using 80 nm citrate capped gold nanoparticles purchased from Ted Pella. A ligand exchange was performed using 1 mg/mL Amine PEG Thiol (MW = 5000) and were agitated for 12 h. The nanoparticle suspension is then centrifuged three times at 600 g, the supernatant is removed, and resuspended in doubly distilled water. Electrostatic adherence of the negatively charged heparin to the positively charge amine on the surface of the gold nanoparticles was accomplished by adding 2 mg/mL of heparin to the colloidal suspension. The nanoparticles were agitated for 12 h, centrifuged three times at 600 g, as the supernatant was removed each time and resuspended in doubly distilled water. A competition assay was developed using collagen-gold nanoparticle conjugates, and incubating with a sample containing heparin for 15 min. After this, 80 nm heparin coated gold nanoparticles were added to the solution. Immediately after the addition of 80 nm heparin coated gold nanoparticles, a UV-Visible measurement was taken. Samples were left alone for 30 min, and a final UV-Visible measurement was taken. Kinetic measurements were carried out as a function of time by first incubating the sample with a given concentration of heparin. Following this, the 80 nm heparin-coated gold nanoparticles were added to the system and the LSPR spectra was monitored over time.

## 3. Results and Discussion

Negatively charged gold nanoparticles have been reported to have a high affinity for collagen [26], most likely due to the abundance of positively changed lysine residues throughout the collagen fibrils. Therefore, the addition of negatively charged citrate capped gold nanoparticles resulted in gold nanoparticle decorated collagen fibrils. Gold nanoparticles were synthesized by applying the reverse Turkevich method [25] to generate monodisperse particles by using a molar ratio of 1:20.8 of HAuCl_4_ generating a diameter of 10.2 ± 2.7 nm, see Figure S1. The collagen-gold nanoparticle conjugates were formed by adding the citrate capped gold nanoparticles to a collagen fibril solution at a 3:5 ratio, followed by 30 min incubation period. 

**Figure 1:**
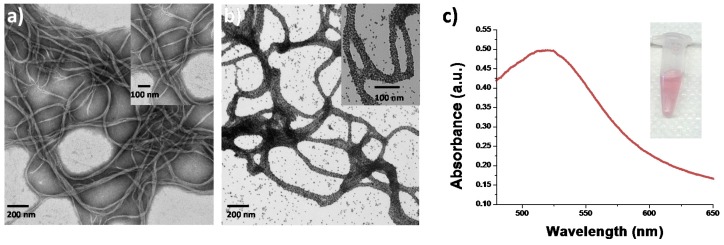
Transmission electron microscopy (TEM) of native collagen fibrils, (**a**); and collagen fibrils coated with gold nanoparticles, (**b**); absorbance spectrum of collagen-gold nanoparticle conjugates, (**c**).

The collagen-gold nanoparticle conjugates were characterized using transmission electron microscopy (TEM). As shown in Figure 1, it was observed that the gold nanoparticles were indeed interacting with the surface of the collagen protein and that fibrils of similar diameter and length were formed in the gold nanoparticle-collagen conjugates. The conformational stability of collagen coated with gold nanoparticles was assessed through circular dichroism spectroscopy (CD) to evaluate the structural viability of collagen fibrils that have been coated with metallic nanoparticles. The CD spectrum of collagen in the native state is indicated by a maximum positive peak around 220 nm and a maximum negative peak around 195 nm [27]. Comparison of the CD spectrum of native state collagen and collagen coated with gold nanoparticles indicates that the gold nanoparticles are not significantly altering the secondary structure of the protein, see Figure S2. No significant change in secondary or tertiary structure of the collagen-nanoparticle conjugates implies that there is a good possibility these conjugates retain the biological activity of native collagen. 

### 3.1. Glucose Detection 

**Figure 2:**
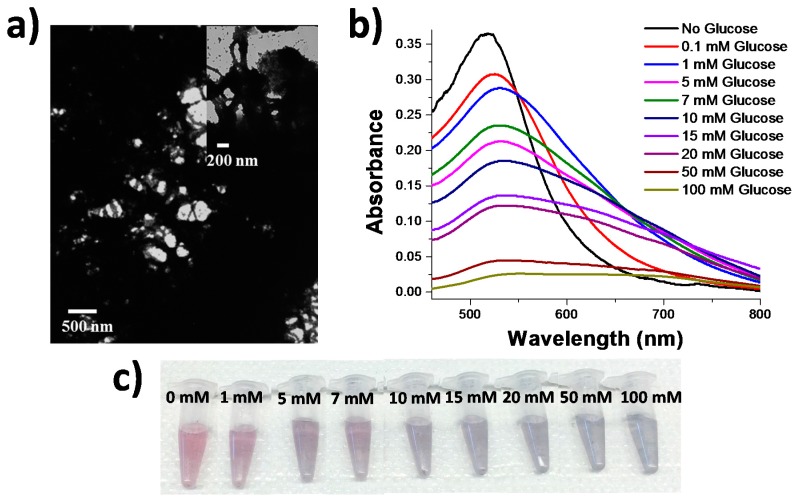
Transmission electron microscopy (TEM) of collagen-nanoparticle scaffolds that have been cross-linked using 10 mM glucose (**a**); spectra of the collagen-nanoparticle arrays with glucose concentrations ranging from 0.1 mM to 100 mM (**b**); the corresponding images of solutions containing the collagen-gold nanoparticle arrays with different concentrations of glucose indicating that simple, colorimetric detection is possible (**c**).

Individuals with diabetes are unable to self-regulate their blood glucose levels, which can lead to serious health complications if not properly monitored [28]. Therefore, it is critical for individuals with diabetes to monitor their blood glucose levels before and after meals in order to maintain healthy levels. Thus, having a sensor that detects glucose in the physiological range in a fast, inexpensive manner has vast clinical significance for hospitals, over-the-counter tests, and point-of-care diagnostic platforms. Previous reports have examined the interactions between glucose and the amino acid residues, lysine and arginine, to form covalent cross-links called glucosepane without the presence of an enzyme [29], both in vivo and in vitro. These products are often referred to as advanced glycation end products (AGEs), and form in vivo when the concentration of glucose exceeds a healthy limit greater than 10 mM. We exploited glucosepane formation to develop a colorimetric sensor for glucose by combining the functional properties of the protein collagen, and combined this with the optically responsive properties of gold nanoparticles. This was achieved by combining the collagen-nanoparticle scaffolds with glucose, and incubating them in a water bath at 35 °C which initiated the formation of aggregates that were observed through TEM. Comparison of the non-aggregated collagen-nanoparticle conjugates, Figure 1a, to collagen gold nanoparticle scaffolds that were cross-linked with 10 mM glucose, Figure 2a, showed a significant difference in the structure of the conjugates. The conjugates that were exposed to 10 mM glucose exhibit densely aggregated fibrils that yielded an LSPR response that is not observed under native collagen conditions; mainly the formation of a largely red-shifted peak ~635 nm, indicative of a substantial amount of plasmonic coupling. This plasmonic coupling observed in the LSPR spectrum is most likely attributed to the formation of the glucosepane linkages pulling the nanoparticle-decorated collagen fibrils into closer proximity to one another. 

**Figure 3:**
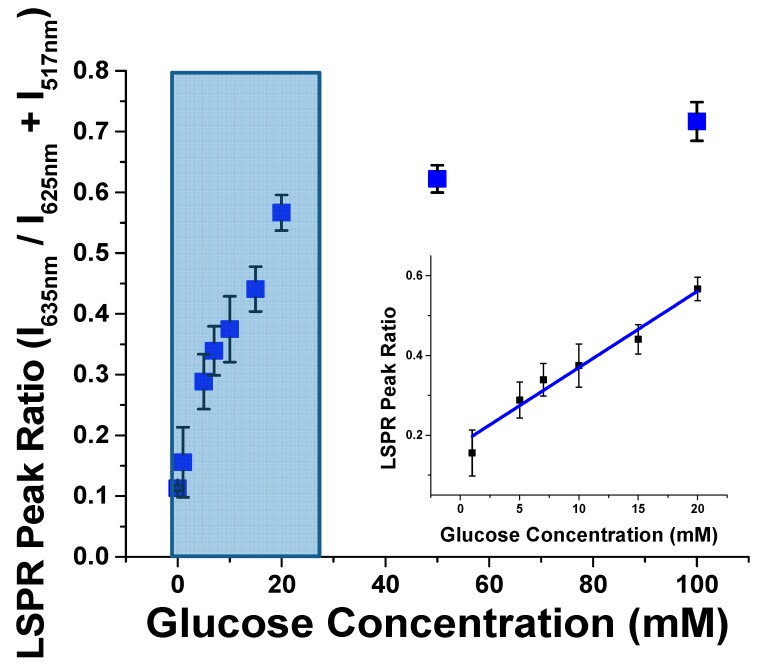
The LSPR change with respect to glucose concentration follows a trend that is consistent with the Langmuir model of binding to a surface. The linear range of detection is depicted in the inset and falls between 1 and 20 mM glucose.

The formation of these cross-links were further characterized both spectroscopically and colorimetrically by an apparent change from the initial collagen-nanoparticle conjugate that appeared pinkish red to the naked eye at an LSPR wavelength of 517 nm, Figure 2b,c. The addition of 1 mM concentrations of glucose resulted in a slightly more purple color than the initial collagen-nanoparticle conjugate appeared. As the concentration of glucose increased to 5–10 mM glucose, a more dramatic color change is observed from red to purple. Once the glucose concentration in solution was greater than 10 mM glucose, the color appeared to turn a purple grey color, and grew increasingly transparent and grey at the highest concentrations of 50 and 100 mM glucose as a result of excessive aggregation and precipitation by glucose. These changes are significant because standard healthy blood glucose levels, 3–7 mM, are distinguishable by eye from elevated concentrations, above 10 mM, and concentrations that are physiologically depressed, below 3 mM. A spectral analysis of the samples revealed that spectral changes occur as low as 1 mM of glucose, as the appearance of a second peak in the infrared region of the spectrum ~635 nm. By increasing the concentration of glucose present in the system, coupling was observed to a greater extent by the larger ingrowth of a second peak ~635 nm that is a result of plasmonic coupling between the AuNPs that coated the collagen fibrils. Spectral data was analyzed by first normalizing the total intensity of all spectra and then fitting the data to two gaussians, one at 517 nm and the other at 635 nm. The normalized intensity of the ~635 nm gaussian was plotted with increasing glucose concentration (Figure 3) and it displays a linear trend between 1 and 20 mM glucose, saturating at concentrations above 20 mM glucose. The system produced a limit of detection of 0.18 mM, which is quite low for a simple colorimetric glucose sensor and potentially useful for patients with diabetes. 

To determine the duration of cross-linking, changes in LSPR wavelength were monitored over time through the addition of 10 mM glucose, see Figure S4. The analyzed spectra were similar to the results in Figure 3, in which two peaks were identified and used to fit the spectra, and the normalized peak intensity of the ~650 nm peak was monitored with time. The changes plateaued by 60 min of incubation in a water bath at 35 °C, and the plot indicated that the majority of the cross-linking effects of glucose occur within the first half hour. These timescales may still be of considerable length for a clinical setting, and further optimization of the assay to reduce the time required for reaction would likely include the addition of crowding agents, such as PEG, and increasing the heat. It is also important to note that the glucosepane cross-linking is covalent and therefore not reversible. Thus, the relatively inexpensive collagen-nanoparticle samples would be ideally suited for one-time use. 

**Figure 4:**
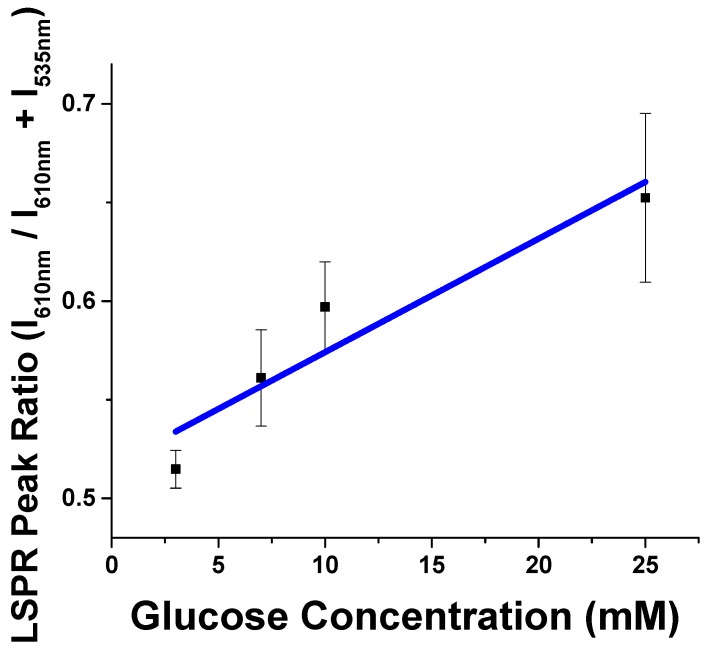
Collagen-nanoparticle conjugates tested with increasing concentrations of glucose in 50% mouse serum. A linear trend is observed in the concentration range of 3–25 mM glucose.

Lastly, to determine the effects of the collagen-gold nanoparticle conjugates in complex solution, glucose cross-linking was tested in 50% mouse serum to determine how this would affect the cross-linking properties. Conjugates were tested in 50% mouse serum against 0, 3, 7, 10 and 25 mM glucose, see Figures 4 and S9. When no glucose was present in the conjugate serum solution, the LSPR spectra could be fit to a single peak ~535 nm. As the concentration of glucose increased in the conjugate serum mixture, an LSPR peak ~610 nm grew in, providing a linear range of detection from 3 to 25 mM glucose which shows clinical significance by showing selectivity and sensitivity in the health relevant range. Thus, protein-nanoparticle conjugates show great promise as selective biosensing species, even in the presence of complex media.

### 3.2. Heparin Detection

In order to demonstrate the versatility of these protein-nanoparticle conjugates, the same conjugates were used to detect a different biological target, heparin. Heparin is an injectable anti-coagulant that is used to treat and prevent blood clots in arteries and veins, and is used during surgery to reduce the risks of blood clots forming. Hence, monitoring heparin concentrations in patients has vast clinical significance [30] for both surgery and patient care. This glycosaminoglycan (GAG) molecule has been shown to interact with a variety of proteins in the extracellular matrix including type 1 collagen [31]. Previous studies have mapped out the heparin binding sites on collagen using heparin functionalized gold nanoparticles on collagen monomers and fibrils [31]. 

Using the known interactions between collagen and heparin, a competition assay to detect heparin with inverse sensitivity was developed using a two-step method. The initial step incubated the collagen-gold nanoparticle arrays with the sample concentration of heparin for 30 min, followed by incubation with 80 nm heparin coated gold nanoparticles. When no heparin is present in the sample, all of the heparin binding sites are available for the 80 nm heparin coated AuNPs to bind to the collagen nanoparticle scaffold. The binding event resulted in plasmonic coupling between the heparin coated gold nanoparticle and the gold coated collagen scaffold and a large red-shifted LSPR peak. When heparin is present in the sample in excess, the heparin binding sites on the collagen-nanoparticle scaffold are occupied; therefore, the addition of heparin coated gold nanoparticles resulted in little to no shift from the initial plasmon resonance. 

**Figure 5:**
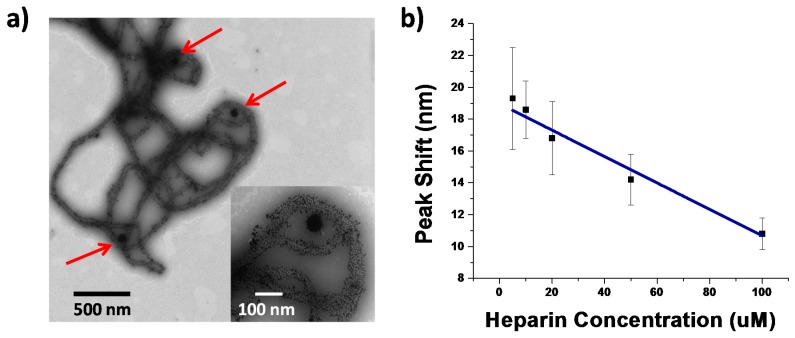
TEM characterization of the interactions between collagen-nanoparticle conjugates and 80 nm heparin coated gold nanoparticles (**a**); the regions in which the 80 nm particles are binding directly to the collagen fibrils are highlighted with red arrows. LSPR shift induced by the binding of the 80 nm heparin coated gold nanoparticles in a competition assay with increasing concentrations of heparin (**b**); since more heparin coated nanoparticles bind when the collagen is exposed to lower concentrations of heparin, the largest LSPR shifts are observed at low heparin concentrations.

These interactions between heparin functionalized gold nanoparticles and collagen-nanoparticle conjugates were observed through TEM, confirming the interactions in the presence of the gold nanoparticles functionalizing the protein, Figure 5a. The interactions were also observed through LSPR shifts toward the red as a result of heparin functionalized gold colloids binding to the collagen-nanoparticle conjugates (Figure S6a). However, when the system has been saturated with heparin in solution, no LSPR shift is observed due to plasmonic coupling (Figure S6b). This indicates that, under saturating heparin conditions, the majority if not all heparin binding sites available on collagen are occupied, and result in no plasmonic coupling between the two species. Additionally, TEM images were taken to further confirm this lack of interaction when the collagen-nanoparticle constructs are saturated with heparin, and indeed no binding was observed between the 80 nm heparin-coated gold nanoparticles with collagen (Figure S8). When there is no free heparin present to bind collagen, the addition of 80 nm heparin functionalized gold colloids are free to bind the nanoparticle-conjugates resulting in plasmonic coupling. Figure 5b shows linear detection through plasmonic coupling of the heparin coated gold nanoparticles and the bionanoparticle scaffolds of heparin are observed from 5 to 100 μM, which lies in the relevant range for monitoring heparin concentrations both during surgery, 17–67 μM, and the upper end of postoperative and long term care, 1.7–10 μM [32]. Similar to glucose detection, the spectral and colorimetric changes occur within 30 min of adding the heparin-coated nanoparticles, see Figure S7. 

## 4. Conclusions

Harnessing the versatile properties of the extracellular matrix protein collagen, novel, label-free, plasmonic coupling detection of specific biological ligands was observed with a single substrate. This proof of concept collagen-nanoparticle scaffold showed versatility through monitoring the interactions of two substrates, glucose and heparin, in the physiologically relevant range. Selectivity was then demonstrated through quantitative measurements of glucose in 50% mouse serum. Such conjugates show great promise regarding simple colorimetric sensing in a selective, yet versatile manner, with improved biological compatibility.

## Supplementary Materials

The following are available online at http://www.mdpi.com/1424-8220/17/2/378/s1. Detailed information containing control experiments, structural characterization, and kinetic measurements.
