# Supplementary material for: Collagen-Gold Nanoparticle Conjugates for Versatile Biosensing
Source: Sensors (Basel). 2017 Feb 15;17(2):378. doi: 10.3390/s17020378 (PMC5335965; doi:10.3390/s17020378)
Supplement: Supplementary file 1 [file sensors-17-00378-s001.pdf]

Supplementary Materials: Collagen-Gold Nanoparticle Conjugates for Versatile Biosensing

Sarah Unser, Samuel Holcomb, ReJeana Cary and Laura Sagle

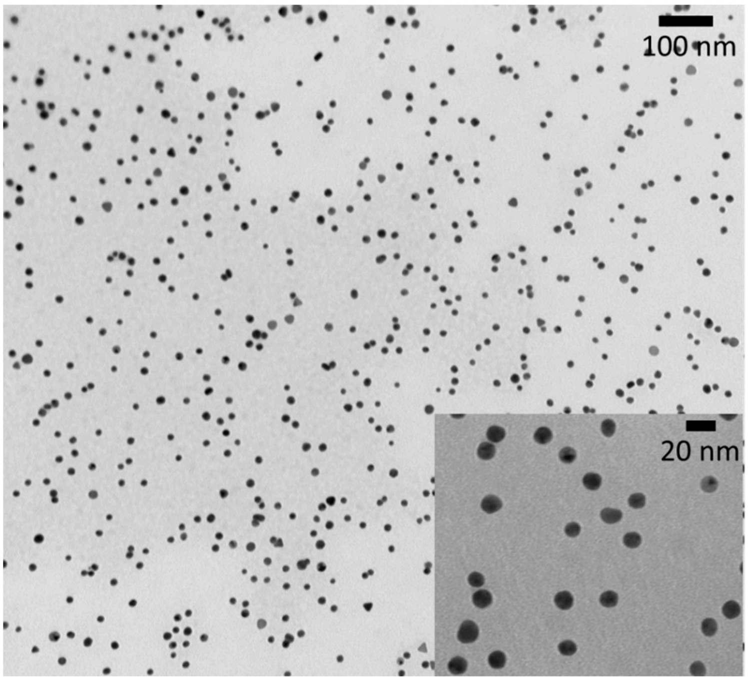

(**a**)

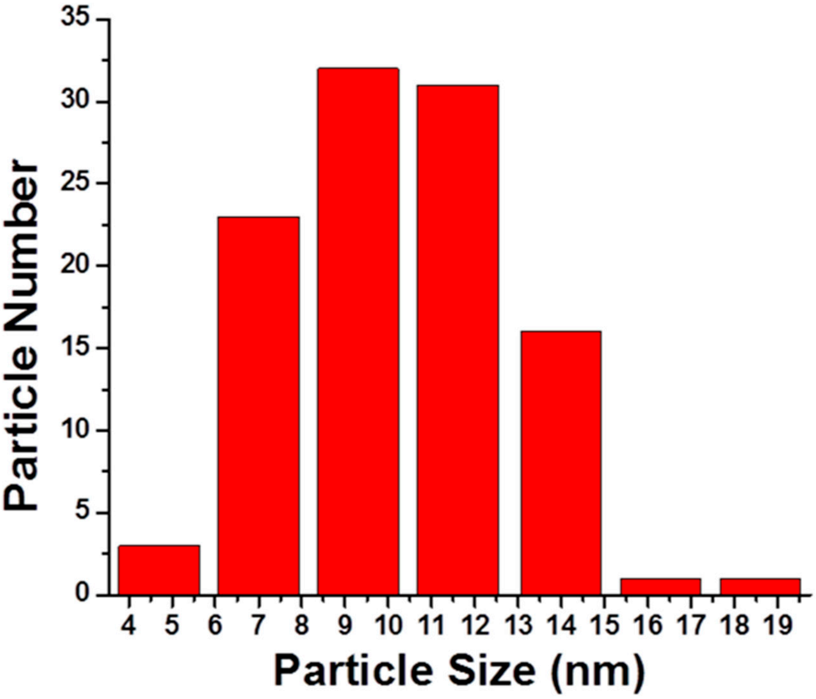

(**b**)

Figure S1. (**a**) Transmission electron microscopy of free citrate capped gold nanoparticles that are formed using the reverse Turkevich method (**b**) histogram of the gold nanoparticles formed with the reverse Turkevich method. The average size of the nanoparticle is 10.2 ± 2.7 nm after analyzing 107 particles.

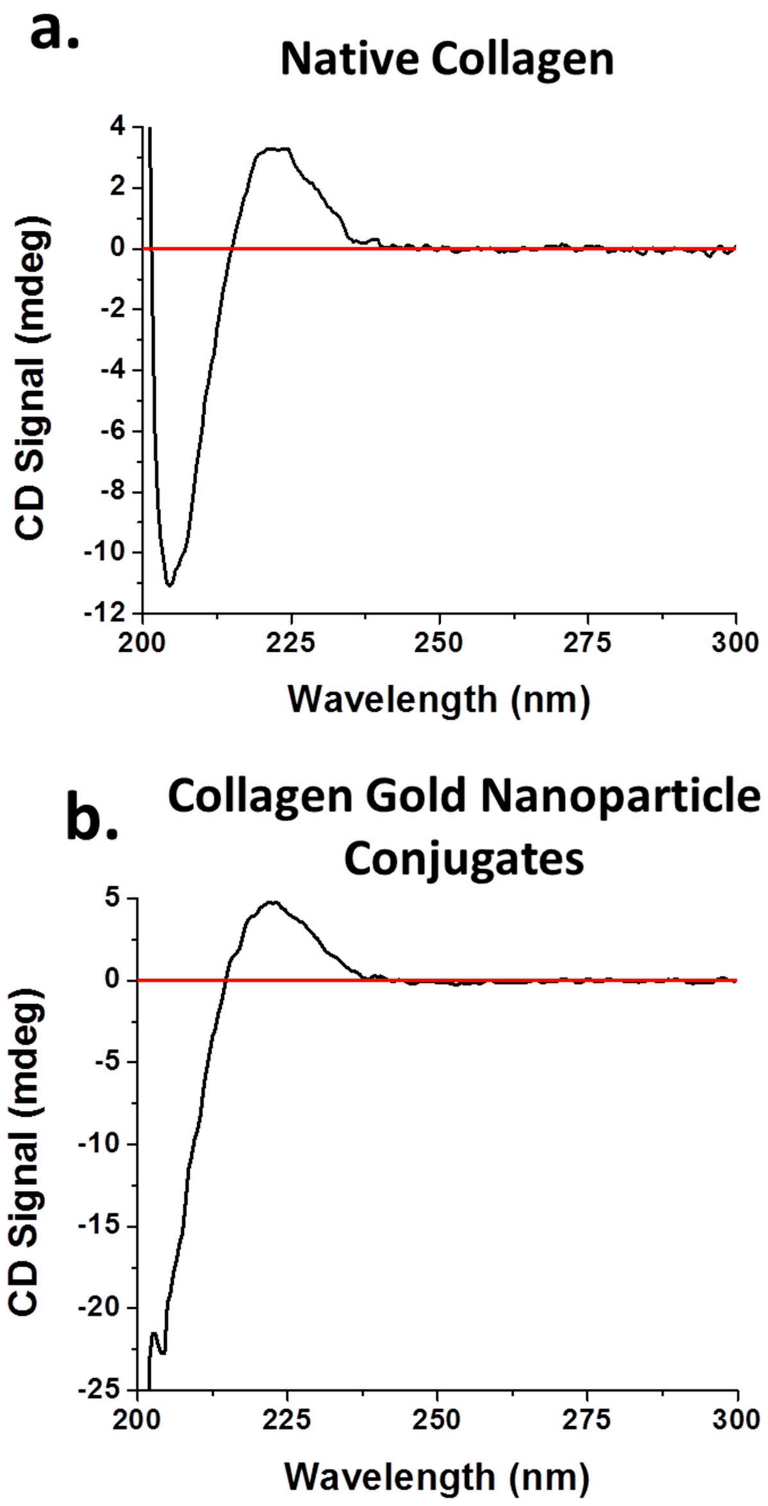

Figure S2. Circular dichroism spectroscopy of (**a**) native collagen fibrils and (**b**) collagen-gold nanoparticle conjugates. The spectra imply the overall structure of the collagen is not disturbed by nanoparticle addition.

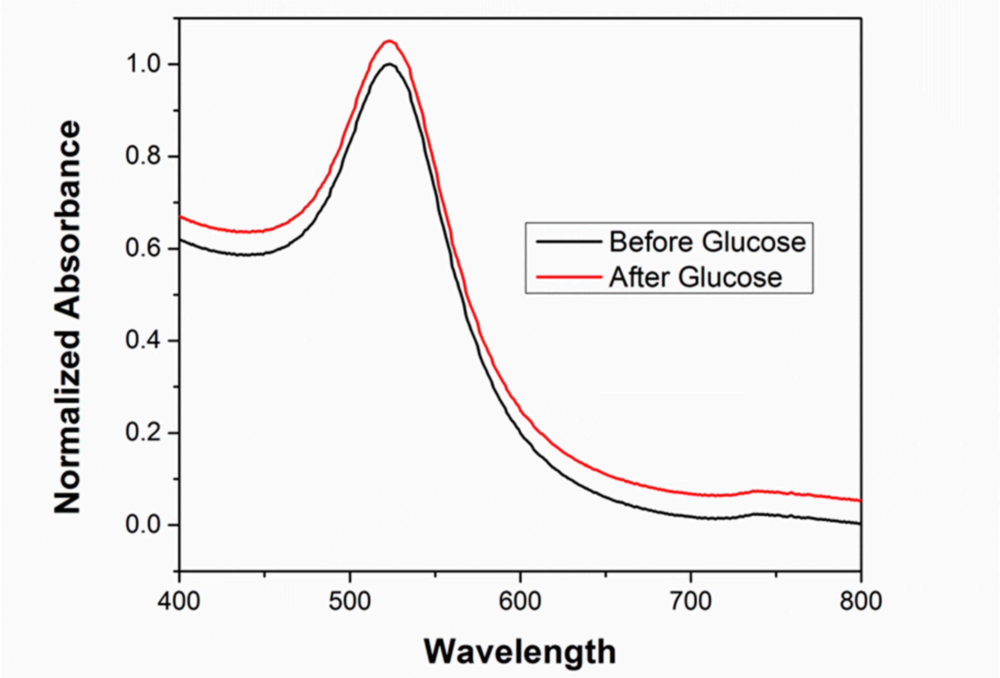

Figure S3. Citrate capped gold nanoparticles before the addition of glucose and after glucose incubation showing there are no nonspecific interactions between glucose and the citrate capped gold nanoparticles.

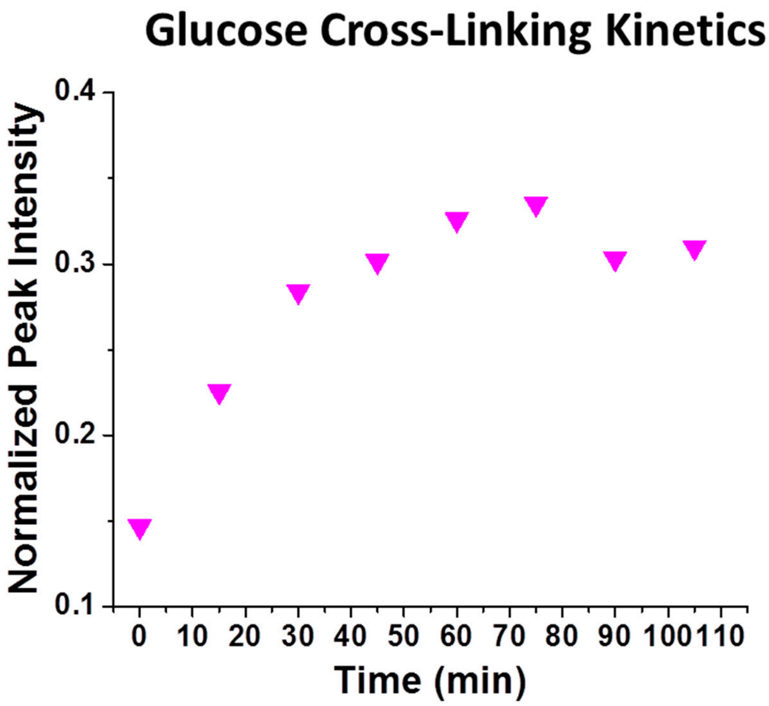

Figure S4. Ingrowth of the LSPR peak due to cross-linking as a result of 10 mM glucose over time. The ingrowth occurs most rapidly within the first 40 min, and begins to level off around 60 min.

a. **DLS of 80 nm Ted Pella citrate capped gold nanoparticles**

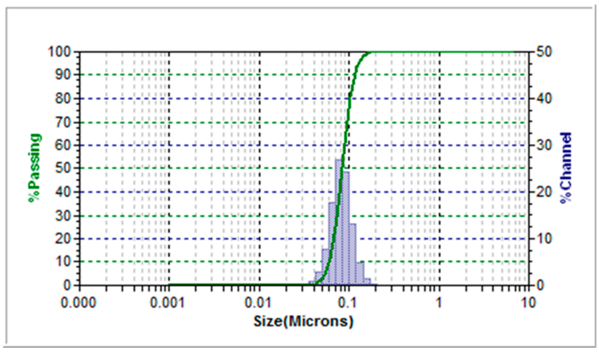

Peaks		
Dia(um)	Vol %	Width
0.0827	100.00	0.0416

b. **DLS of 80 nm Ted Pella gold nanoparticles with heparin**

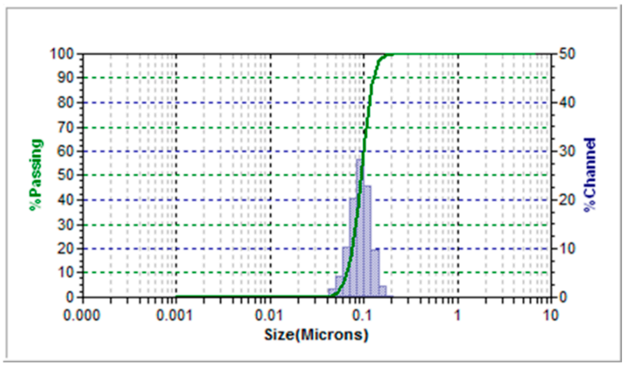

Peaks		
Dia(um)	Vol %	Width
0.0934	100.00	0.0454

Figure S5. Dynamic light scattering of (**a**) citrate capped 80 nm gold colloids that were purchased from Ted Pella and (**b**) 80 nm gold colloids that are functionalized with heparin. The increase in diameter is indicative of heparin functionalization.

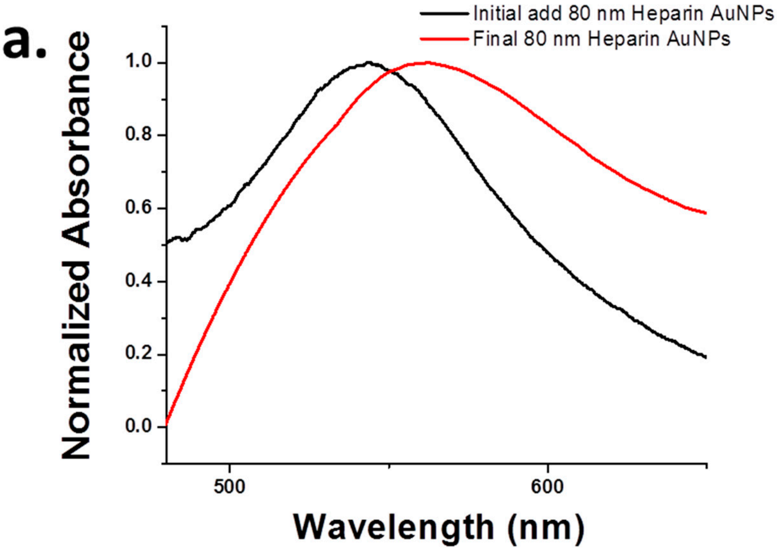

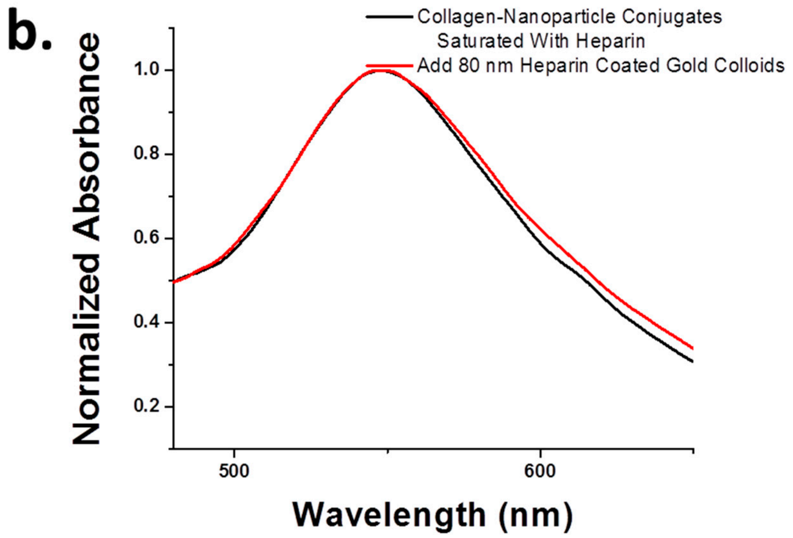

Figure S6. LSPR shifts for (**a**) collagen-nanoparticle conjugates with 80 nm heparin functionalized gold colloids show a 22 nm shift due to plasmonic coupling between the colloids and nanoparticle conjugates and (**b**) heparin saturated collagen-nanoparticle conjugates with 80 nm heparin functionalized gold colloids yielding little to no plasmonic change.

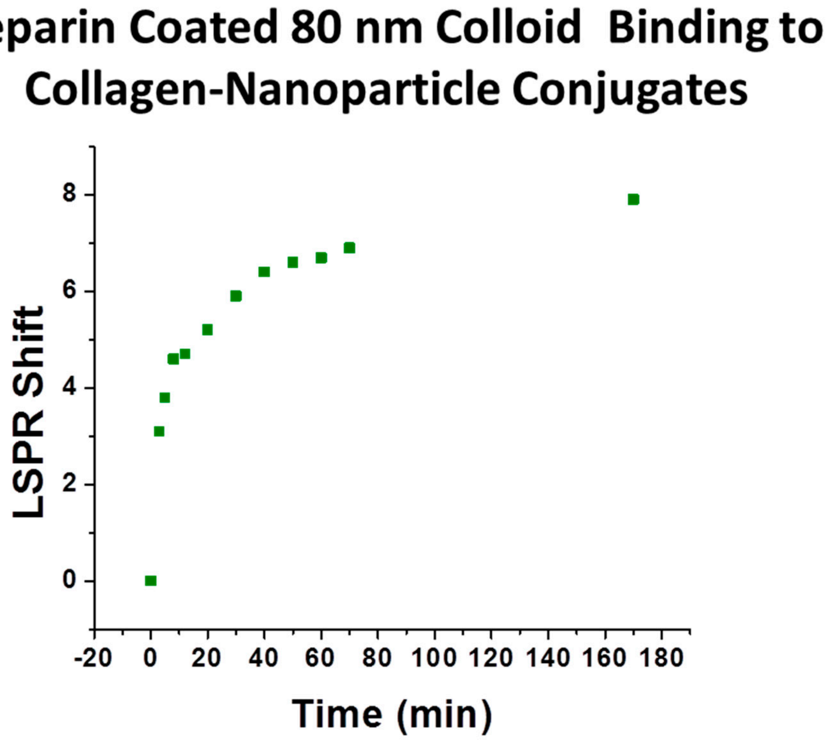

Figure S7. Heparin coated 80 nm gold nanoparticles binding to collagen-nanoparticle conjugates interactions over time. The largest changes are observed within the first 30 min.

Adding Heparin-Coated Gold Nanoparticles To Heparin Saturated Collagen

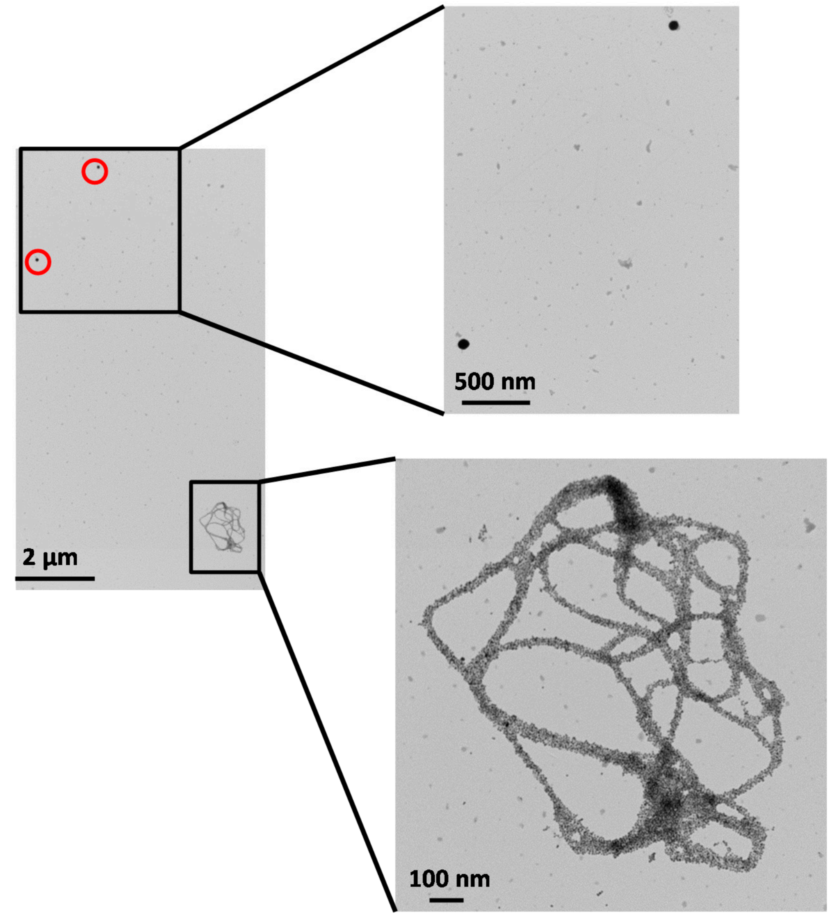

Figure S8. TEM images of mixing heparin-saturated collagen-nanoparticle constructs, in which 500 uM of heparin had been added, with 80 nm heparin-coated gold nanoparticles. The images to the right are zoomed in the regions indicated in the zoomed out image on the left. The images indicate the 80 nm heparin-coated gold nanoparticles added to solution do not interact with the collagen that is already coated in heparin.

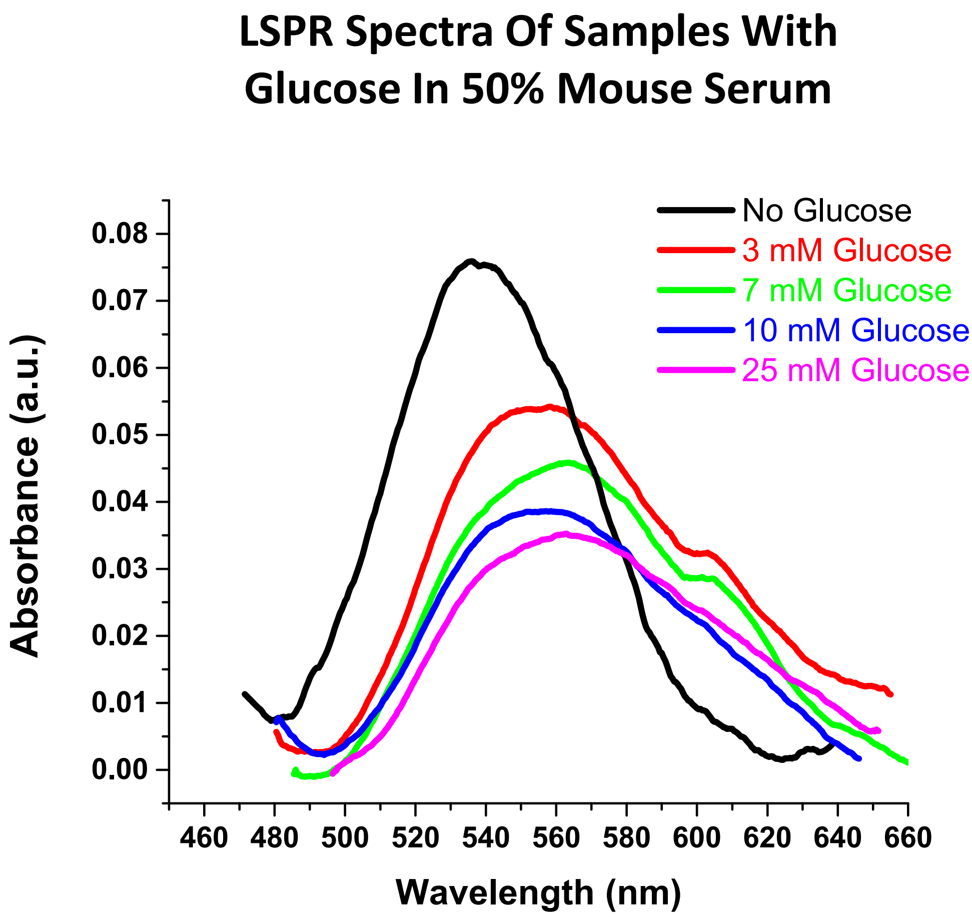

Figure S9. Collagen-nanoparticle conjugates are tested in 50% mouse serum with increasing amounts of glucose added to the samples. Similar to the samples without serum, a red-shifted peak ~610 nm grows in as the glucose concentration increases.
